# Direct somatic embryogenesis, genetic fidelity assessment and GC-MS analysis of regenerated *Crinum asiaticum* L. Plants

**DOI:** 10.1186/s12870-025-07327-7

**Published:** 2026-01-06

**Authors:** Yashika Bansal, A. Mujib, Jyoti Mamgain, Shruti Grover, Yaser Hassan Dewir, Árpád Székely

**Affiliations:** 1 1Cellular Differentiation and Molecular Genetics Section, Department of Botany, Jamia Hamdard, New Delhi, India; 2 Department of Plant Production, College of Food and Agricultural Sciences, King Saud University, Riyadh, 11451 Saudi Arabia; 3 Research Centre for Irrigation and Water Management, Institute of Environmental Sciences, Hungarian University of Agriculture and Life Sciences, Anna-Liget str. 35, 5540 Szarvas, Gödöllő, Hungary

**Keywords:** Flow cytometry, Molecular marker analysis, Phytocompounds, Scanning electron microscopy, Somatic embryogenesis, Spider lily

## Abstract

**Background:**

*Crinum asiaticum L.* is an important reservoir of phytocompounds containing galanthamine, lycorine, tazettine and others with diverse pharmacological uses. Due to high commercial demand for these promising compounds in pharmaceutical sector, an efficient in vitro micropropagation protocol optimization study was conducted via direct somatic embryogenesis in *C. asiaticum*. The regenerated plants were subject to genetic fidelity assessment; and the phytochemical composition was analysed and compared with donor plants. In this investigation, the bulb-scales were used as explants onto media containing different PGRs for various regeneration processes.

**Results:**

In media containing BAP and NAA, somatic embryos were formed directly on bulb-scale explant surfaces with the highest (95.83%) being at MS medium + 2.7 µM NAA + 4.4 µM BAP. The occurrence of somatic embryos at different stages was confirmed by histological and scanning electron microscopic (SEM) analysis. The embryos were later converted to shoots on 2.2–8.8 µM BAP augmented MS medium, with highest germination percentage of 75 ± 7.22 at 4.4 µM BAP. These regenerated plants were successfully transferred to medium containing NAA, IBA or IAA for rooting and the best rooting response (91.67% rooting frequency, 7.67 mean root numbers/shoot and 7.5 ± 0.6 cm average root length) was noted at 5.4 µM NAA. The plants were transferred to greenhouse with pretty good growth and survival. The genetic fidelity of tissue cultured plants was checked through cytological, flow cytometric and SCoT marker-based PCR technique. The root tips of in vitro raised and mother plants showed 2n = 44 chromosome numbers, and the flow cytometric histograms revealed similar fluorescence peaks with nuclear 2 C DNA content of 31.79 and 31.51pg, respectively, displaying no change in ploidy level. Six SCoT primers based genetic homogeneity study showed 42 scorable, monomorphic bands, confirming true-to-type regenerated plants. Finally, the GC-MS based metabolite profiling of in vivo and in vitro raised plants were conducted, which exhibited a wide range of bioactive compounds like tazettine, squalene, gamma-tocopherol, beta-sitosterol, glycidyl palmitate, glycidyl oleate of pharmacological significance.

**Conclusions:**

The current study presents an effective method for genetically stable clonal propagation of *C. asiaticum* for extraction of compounds like tazettine, squalene, beta-sitosterol for pharmaceutical applications.

## Introduction

*Crinum asiaticum* (poison bulb or spider lily) is a perennial bulbous plant possessing white showy flowers and belongs to the Amaryllidaceae family [1]. It is an endemic species to south-eastern part of Asia, now has been widely distributed across continents like America, Africa and Australia [2]. The plant has been long-known for its ornamental value. Different plant parts like bulbs, leaves and roots have frequently been used in traditional medicine to treat wounds, injuries, piles, hemorrhoids, arthritis and polyuria [3]. *Crinum* spp. is a potent source of characteristic alkaloids like lycorine, galanthamine, tazettine, crinine, crinamine possessing a broad range of biological properties such as anti-oxidant, anti-viral, anti-carcinogenicity [4]. Among these compounds, lycorine and galanthamine are of utmost importance. Lycorine is a potential medication that demonstrates protective properties against oxidative damage, ovarian cancer, and cytotoxicity [5]. Similarly, galanthamine is a selectively reversible and competitive inhibitor of acetylcholine esterase and it is a highly recommended drug in treating Alzeihmer’s disease [6].

Owing to over-exploitation, unsustainable collecting methods, urban development and the need to meet the ever-increasing demand of beneficial drugs by pharmaceutical sector, *C. asiaticum* has attracted researchers’ interest. *C. asiaticum* usually propagates by sexual (seed) and asexual (vegetative) methods, but these conventional methods are not sufficient to meet large-scale commercial demand [6]. Therefore, it is necessary to develop an alternative, efficient and high frequency plant regeneration protocol for commercial production of quality *C. asiaticum* plants. The in vitro culture technology allows rapid multiplication and proliferation of microbe free plants under controlled conditions [7]. Somatic embryogenesis is one of the most widely studied approaches for mass propagation, germplasm conservation and genetic transformation of elite plants. Direct somatic embryogenesis, in particular, is regarded an efficient regeneration system for obtaining true-to-type plants as it reduces genetic alteration possibilities [8]. Beside totipotency reconfirmation, the technique possesses advantages over other available in *vitro* methods, such as in faster, large-scale, low-cost production, high synchronicity and in automation [9].

The in vitro tissues/cultures are however, under stress due to plant growth regulators (PGRs) exposure, high osmotic pressure, prolonged subculturing, which promote somaclonal variations [10]. Thus, it is necessary to assess the genetic stability of micropropagated plantlets [11]. Cytological analysis is a useful method to check variation of chromosomal numbers of in vitro obtained plants with respect to in vivo [12]. In recent years, the flow cytometry method (FCM) has gained immense popularity in examining ploidy by measuring the relative nuclear DNA content of tissue cultured plant [13, 14]. Additionally, the DNA based molecular markers such as random amplified polymorphic DNA (RAPD), inter simple sequence repeat (ISSR) and start codon targeted (SCoT) play an important role in validating genetic homogeneity of in vitro raised plants, surpassing the dependency on morphological and physiological traits. SCoT markers have been designed based on DNA sequences flanking the ATG initiation codon [15]. SCoT marker detects genetic variability in regenerants by screening total plant genome with high reproducibility [16] and has been successfully employed in different plants such as *Pisum sativum* L., *Morus alba* L [17, 18]. Gas chromatography-mass spectroscopy (GC-MS) is one of the most widely used analytical techniques for identification and quantification of phytocompounds, present in plant samples [19]. It has been frequently used in detecting bioactive compounds in tissues of several in vitro grown plants [20, 21].

In vitro techniques have been employed for micropropagation of several Amaryllidaceae members like *Lapiedra martinezii* [22], *Narcissus tazetta* [23], *Rhodophiala pratensis* [24]. But very few reports are available on embryogenesis-based plant regeneration in *Crinum asiaticum* [25–27]. The current study aimed to investigate somatic embryogenesis and subsequent plant regeneration in *C. asiaticum* using bulb-scale through histology and SEM analysis. The genetic homogeneity of regenerated plants was also assessed using cytology, flow cytometry and SCoT molecular marker technique. Finally, a comparable secondary metabolite profiling of in vitro and in vivo derived plants was made through a GC-MS based metabolomics approach.

## Results

### Direct somatic embryogenesis

**Fig. 1:**
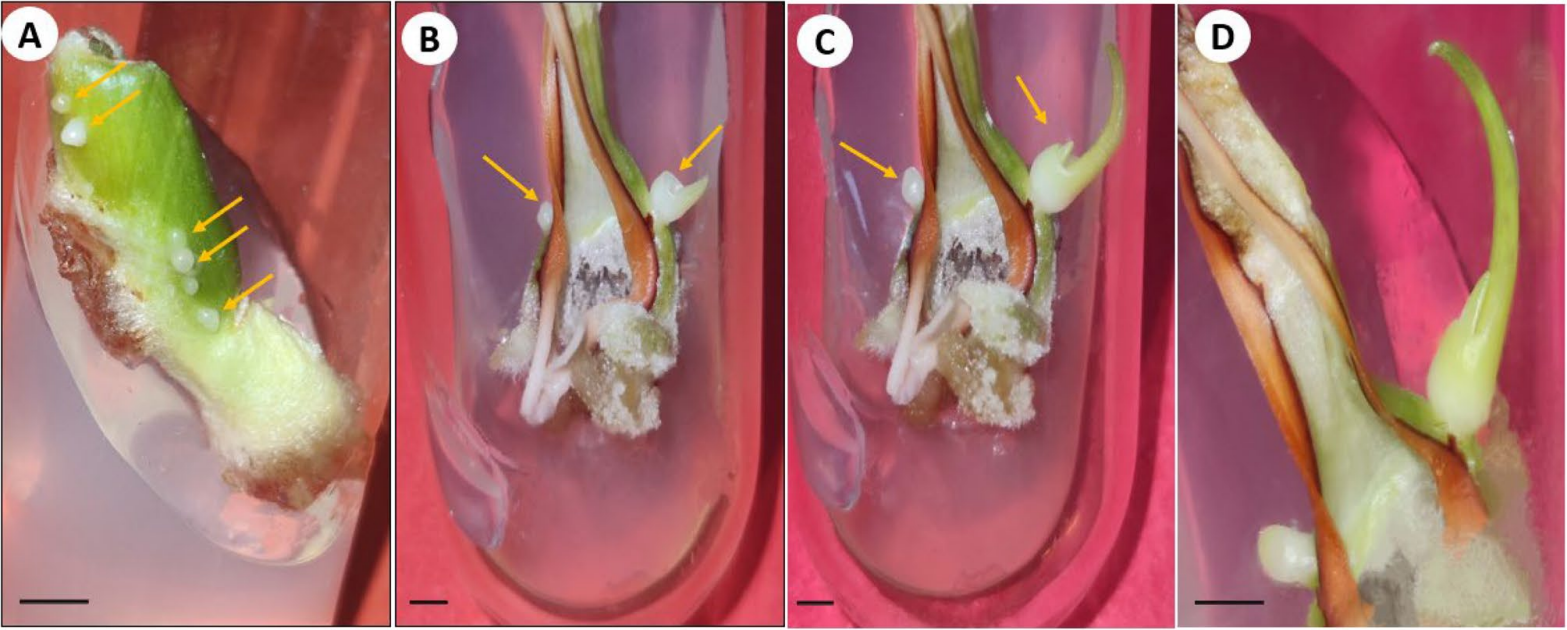
Direct somatic embryogenesis on bulb scale explants of *C. asiaticum* L. on MS (Murashige and Skoog) medium fortified with 4.4 µM BAP (6-benzyl aminopurine) and 2.7 µM NAA (α-naphthalene acetic acid). **A** Emergence of pre-globular embryogenic structures directly on the explant represented by arrow heads (Bar = 0.5 cm). **B** Arrowhead indicating scutellar stage of somatic embryo (Bar = 1.0 cm). **C** Transition of scutellar to coleoptilar stage of somatic embryo (Bar = 1.0 cm). **D** Germination of a healthy somatic embryo on 4.4 µM BAP fortified MS medium (Bar = 0.5 cm)

**Fig. 2:**
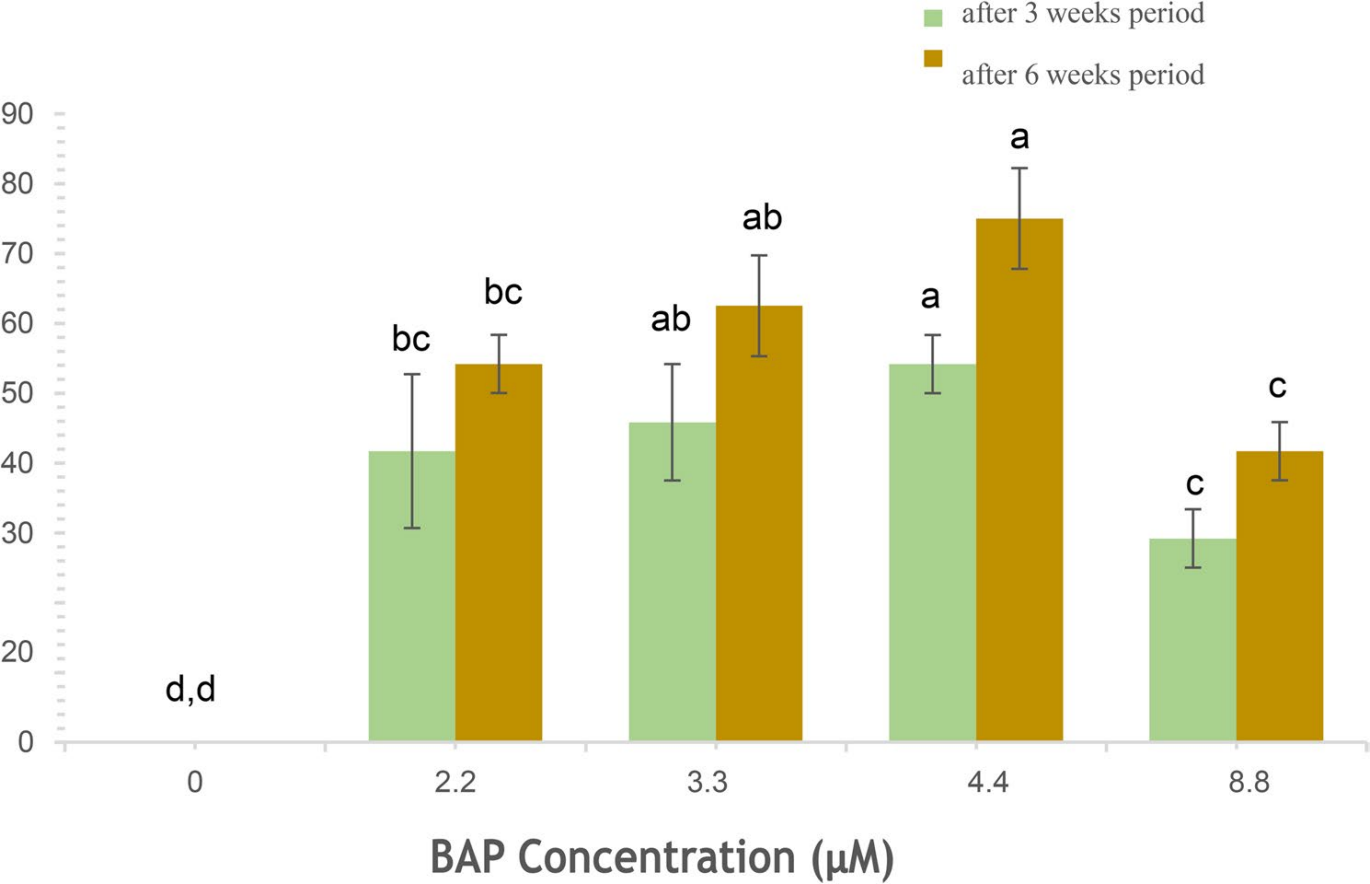
Effect of MS medium augmented with BAP at different concentrations on somatic embryo conversion rate to shoots post three and six-weeks period, respectively. Each value represents the Mean ± SE of three repeated experiments. Mean values followed by the different superscripts in a same column are significantly different from each other according to DMRT at *p* = 0.05 level

The culture of bulb-scales onto MS medium fortified with auxins (NAA, 2, 4-D) and cytokinin (BAP) resulted in somatic embryo emergence on bulb-scale surfaces within 4 weeks of incubation. The somatic embryos emergence was identified visually (Fig. 1A). The NAA and BAP combinations showed higher positive influence on embryo development as compared to the 2, 4-D treatment, used alone (Table 1). The maximum embryogenesis frequency (95.83% ± 4.12) and the mean somatic embryo numbers/explant (8.33 ± 0.33) were observed on 2.7 µM NAA and 4.4 µM BAP added medium. The frequency (8.33 to 58.33%) and somatic embryo numbers (1.33 to 5.33) were, however, low on 2,4-D supplied medium. The somatic embryos at globular stage were white initially, and turned to green at later developmental stages, post six weeks of incubation (Fig. 1B, C). The successful conversion of embryos (Fig. 1D) was achieved on all BAP treatments, with the highest recorded on 4.4 µM BAP and the conversion frequency increased with time (from 54.17 to 75% in three weeks period) (Fig. 2).

**Table 1.**
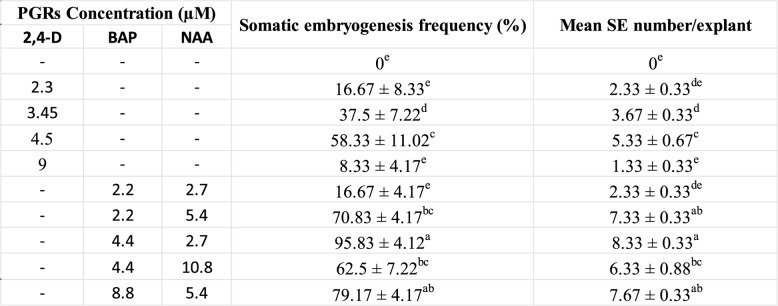
Effect of different concentrations and combinations of PGRs on direct somatic embryogenesis from bulb scale explant of *C. asiaticum* L. after 4 weeks if inoculation

Each value represents the Mean ± SE of three repeated experiments. Mean values followed by the different superscripts in a same column are significantly different from each other according to DMRT at p ≤ 0.05

## Histological and scanning electron microscopy of somatic embryos

**Fig. 3:**
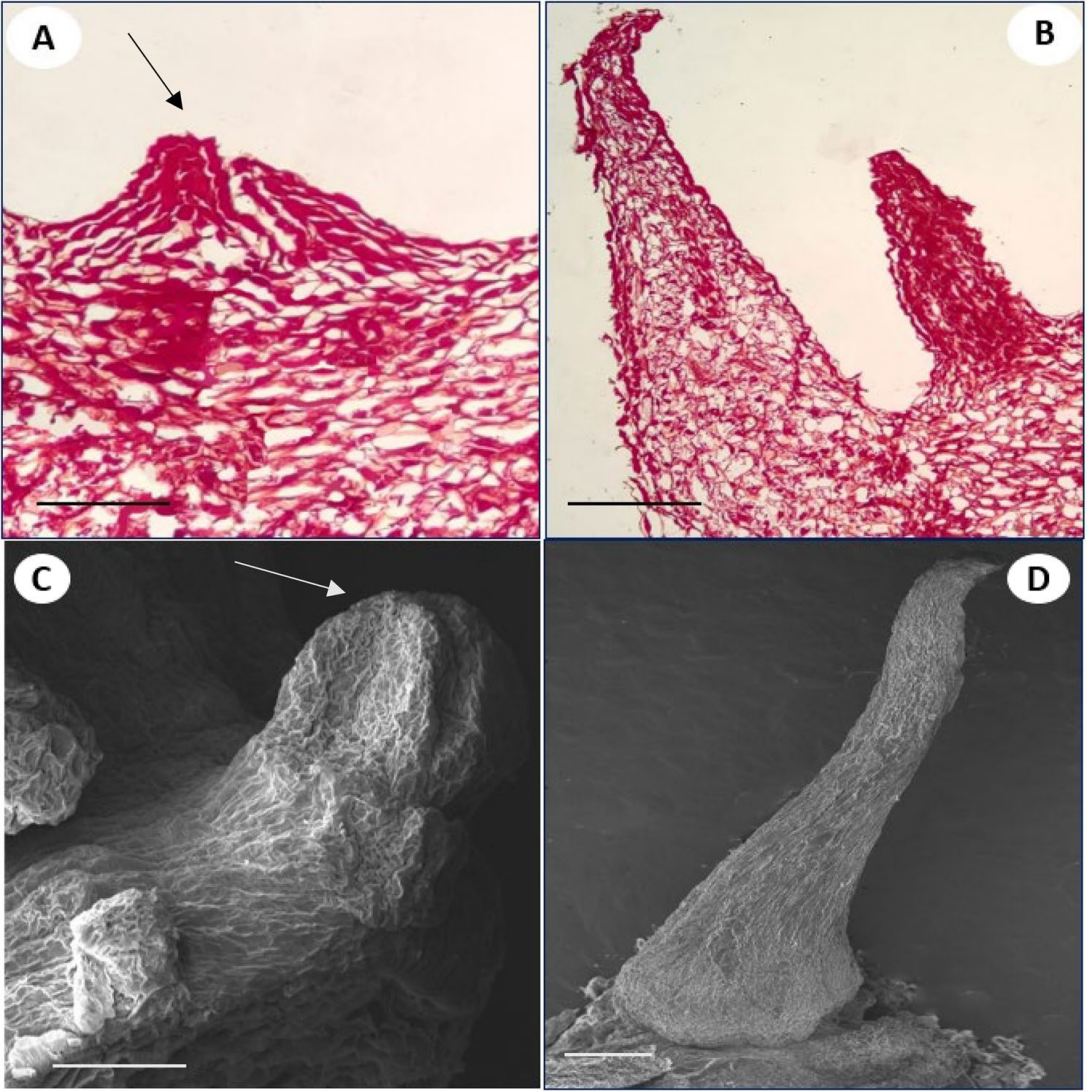
Histological and SEM (scanning electron microscopic) evaluation of formation of direct somatic embryos in *C. asiaticum* L. **A** Pre-globular embryo indicated by arrowhead (Bar = 100 μm). **B** Coleoptilar stage of embryo (Bar = 100 μm). **C** SEM image of early stage of embryo indicated by arrowheads (Bar = 100 μm). **D** SEM image of coleoptilar stage of embryo on scale surface (Bar = 1 mm)

Histological analysis was performed to study the morphology, cellular origin and development of somatic embryos. After 24 days of incubation, the cell aggregates were formed as small protrusions which emerged as pre-globular structure with dense cytoplasm as revealed by longitudinal section (Fig. 3A). The cell division was noticed from the epidermal region which later organised as globular structures. The coleoptilar stage shows a prominent scutellum and coleoptile region (Fig. 3B). To accumulate more information and confirmation of origin, morphology, and other features of embryos, the scanning electron microscopic (SEM) studies of embryo bearing explants were conducted. The SEM investigations revealed the presence of different stages of somatic embryos i.e. scutellar and coleoptilar on the bulb-scale surfaces (Fig. 3C, D).

## Root induction and acclimatization

**Fig. 4:**
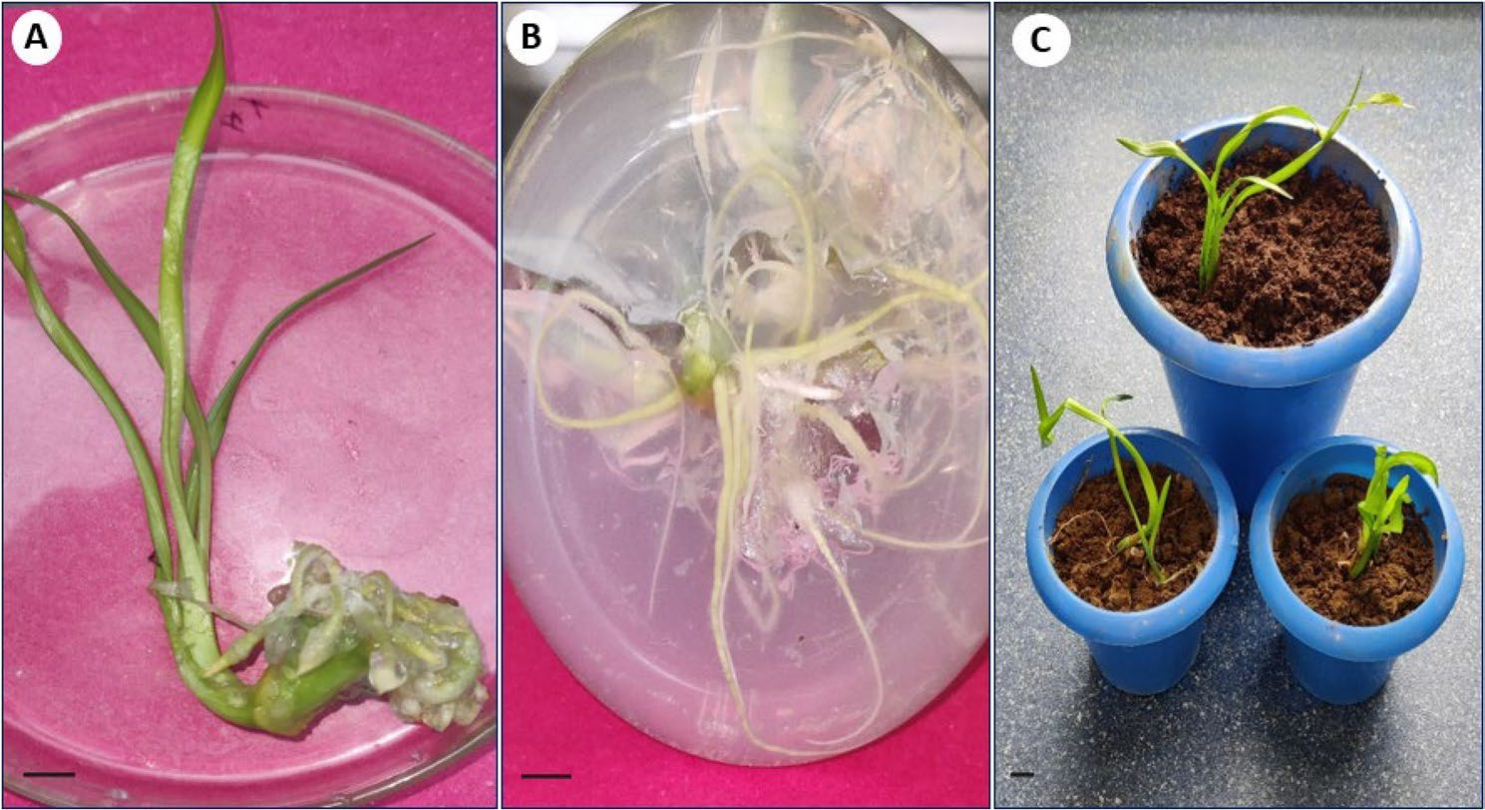
Root induction and acclimatization of tissue cultured raised shoots of *C. asiaticum* L. on 5.4 µM supplied MS medium. **A**, **B** In vitro rooting of shoots (Bars = 1.0 cm), and (**C**) Acclimatized (somatic embryo derived) plantlets in pots (Bar = 3.0 cm)

In vitro regenerated healthy shoots (somatic embryo derived) were transferred to MS medium containing various concentrations of auxins (NAA/IAA/IBA). The influence of PGRs on root induction is presented in Table 2. Out of all tested PGR treatments, NAA treatment showed highest rooting response (70.83–91.67%) as compared to IAA (8-12.5%) and IBA (37.5-54.17%). The best root induction (91.67% ± 8.33) was recorded at 5.4 µM NAA with 7.67 ± 0.33 average roots per shoot and 7.5 ± 0.6 cm mean root length (Fig. 4A, B). Both IBA and IAA showed moderate to little response in rooting, mean root numbers per shoot and mean root length, the least rooting (8.33 ± 4.17%) was observed in 2.9 µM IAA added MS medium with average 1.33 roots per shoot and 3.1 ± 0.4 cm average root length. Afterwards, the regenerated plants were transferred to plastic pots, covered with polythene bags with holes for nearly two weeks, and showed nearly 85–90% survival rate. The regenerated plantlets were grown normally in outdoor conditions (Fig. 4C).

**Table 2.**
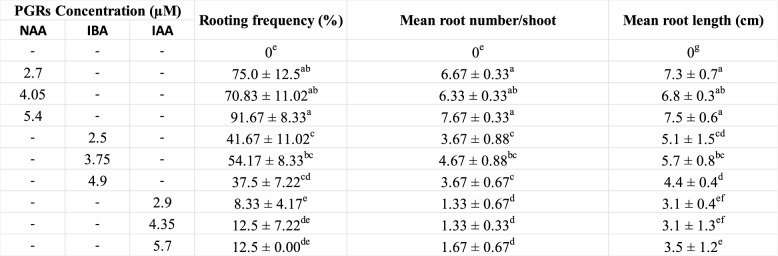
Effect of different concentration of auxins (IAA, IBA and NAA) on *in vitro* rooting of shoots of *C. asiaticum* L. after 4 weeks of inoculation

Each value represents the Mean ± SE of three repeated experiments. Mean values followed by the different superscripts in a same column are significantly different from each other according to DMRT at p ≤ 0.05

## Cytological analysis

**Fig. 5:**
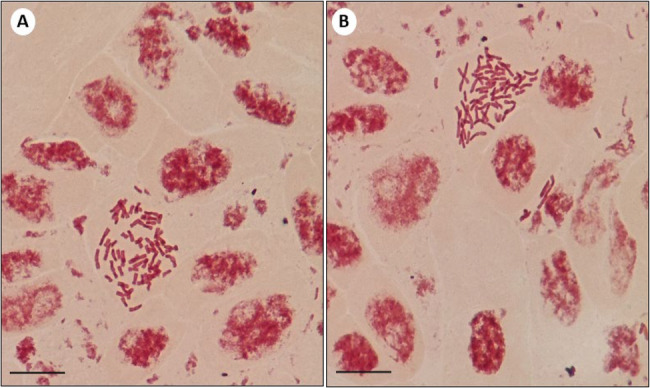
Micrographs of somatic chromosomes in the root tips of (**A**) mother (donor) plant, and (**B**) in vitro regenerated plant of *C. asiaticum* L. displaying equal number of chromosomes (2n = 44). (Bars = 100 μm)

The number of chromosomes from young root tips of in vivo and in vitro (somatic embryo derived) plants were counted to assess the genetic uniformity of regenerated plants. The in vivo and in vitro raised plants were morphologically similar and both displayed normal chromosome numbers. It was observed that both the plants had equal number of chromosomes (2n = 4x = 44) in somatic tissues (Fig. 5A, B), thus confirming genetic uniformity of regenerated samples.

## Flow cytometric study

**Fig. 6:**
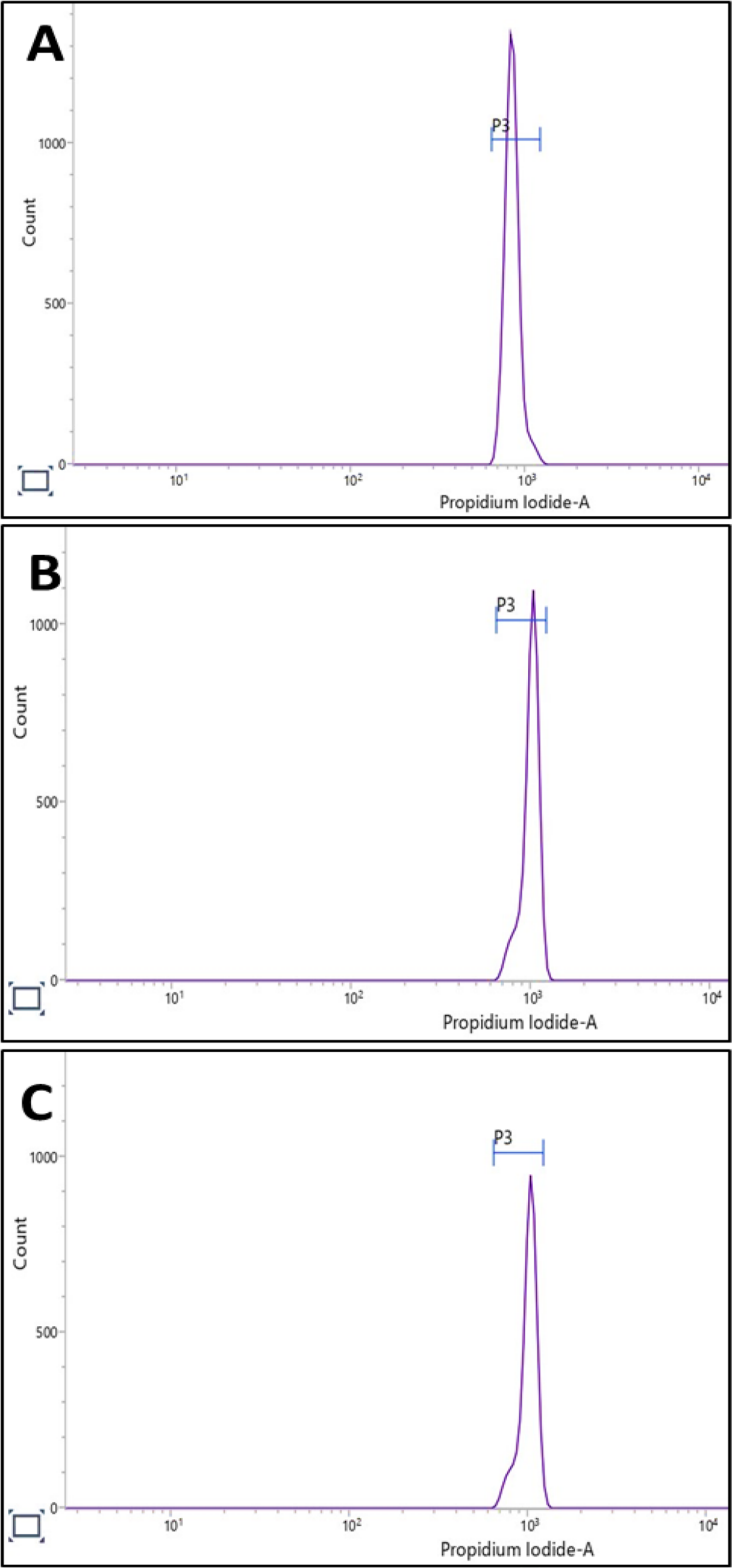
Flow cytometric histograms of the PI-stained nuclei isolated from leaves of (**A**) Standard plant (*Zephyranthes grandiflora*), (**B**) Mother (donor) plant of *C. asiaticum* L., and (**C**) Somatic embryo derived plant of *C. asiaticum* L

To verify the true-to-type nature of somatic embryo regenerated *C. asiaticum*, the flow cytometric approach was used by assessing the ploidy and relative genomic size. In the present study, the 2 C DNA content of mother plant and somatic embryo derived plants were measured. The results revealed the similarities in fluorescence peak intensity of both the samples (Fig. 6B, C), and the 2 C DNA content of in vivo and in vitro (somatic embryo regenerated) plants were measured to be 31.51 and 31.79 pg respectively (Table 3). The estimated genome size of in vivo grown plant was 30,816.78 Mbp and in in vitro plant the genome size was 31,090.62 Mbp. The DNA index of both the samples was found to be in the range of 1.17–1.18 and these values indicated that there were no significant changes in nuclear 2 C DNA content between mother and tissue culture raised *C. asiaticum* plants, thus validating the genetic stability of laboratory grown plantlets.

**Table 3:** Comparison of nuclear 2 C DNA content, genome size and DNA index of somatic embryo regenerated plants and donor (field grown) plants of *C. asiaticum* L

Plant Sample	2 C DNA content (pg)	1 C DNA content (pg)	Genome size (Mbp)*	DNA index**
In vivo plant	31.51 ± 0.01^a^	15.75 ± 0.01^a^	30,816.78^a^	1.17^a^
In vitro derived plant	31.79 ± 0.01^a^	15.89 ± 0.01^a^	31,090.62^a^	1.18^a^

Each value represents the Mean ± SE of three repeated experiments. Mean values followed by the different superscripts in a same column are significantly different from each other according to DMRT at *p* ≤ 0.05 level

*1 pg = 978 Mbp [72]

**DNA index = 2 C DNA content _sample_/2 C DNA content _standard_

### SCoT marker assisted genetic stability analysis

**Fig. 7:**
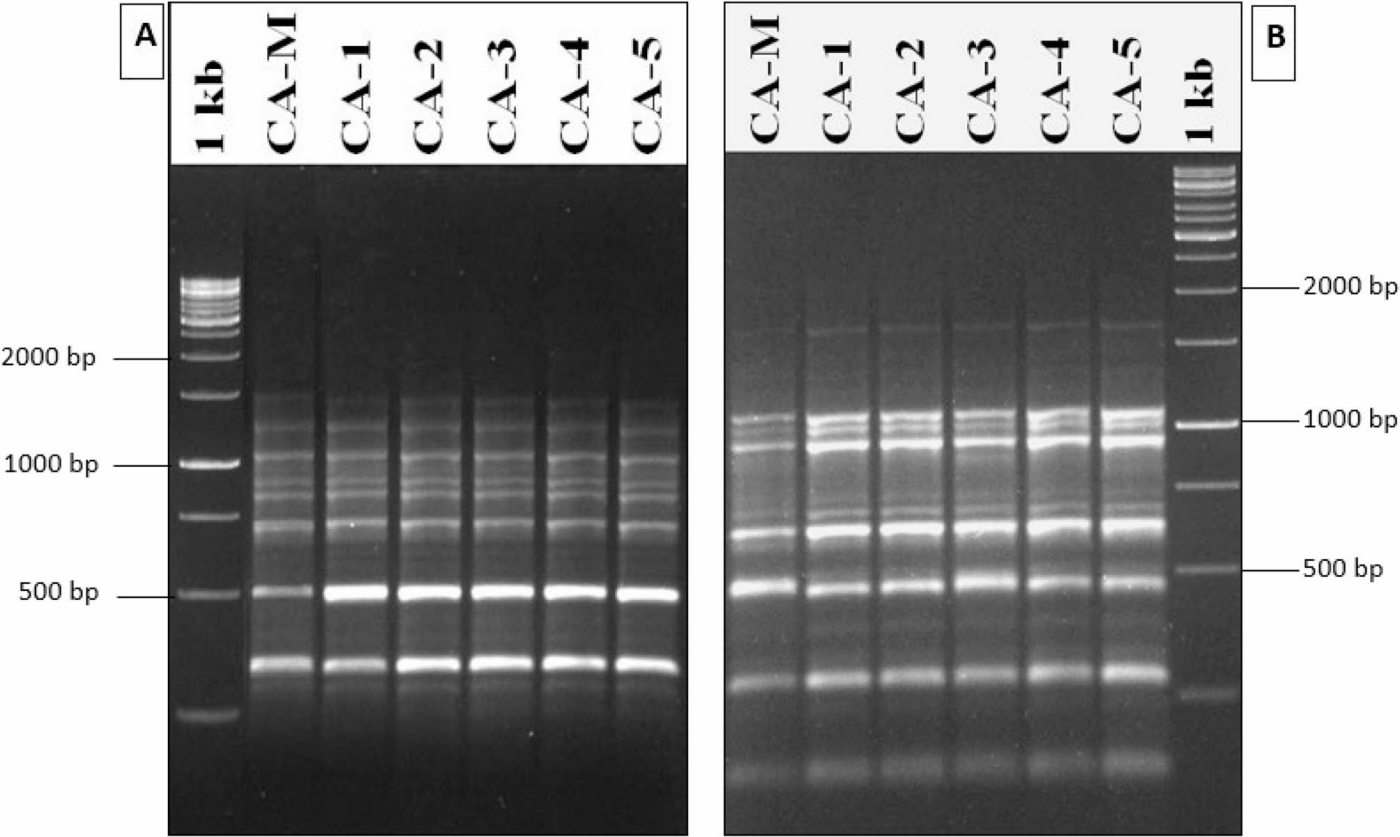
SCoT banding profiles of the mother plant (CA_M) and in vitro derived plants (CA_1 to CA_5) of *C. asiaticum* L. using (**A**) SCoT primer 18, and (**B**) SCoT primer 23

**Fig. 8:**
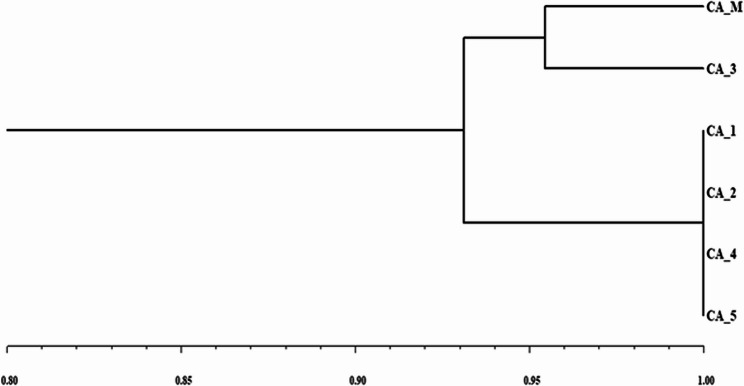
UPGMA dendrogram of SCoT marker analysis showing the genetic relationship between the mother plant (CA_M) and somatic embryo derived plantlets of *C. asiaticum* L

The banding profiles produced by SCoT markers were evaluated to ascertain the genetic homogeneity of somatic embryo derived *C. asiaticum* with respect to their mother plants. A total of 10 SCoT primers were tested, out of which 6 primers (SCoT 3, SCoT 6, SCoT 12, SCoT 18, SCoT 23 and SCoT 33) generated 42 reproducible and clear bands and the size of amplified bands varied from 200 to 1500 bp (Table 4). An average of six bands per primer were scored, all of which were monomorphic, with the highest scorable bands [9] identified from SCoT 3 and SCoT 23 primers and lowest bands [4] from SCoT 12 primer. These amplification patterns revealed no genetic variations between in vitro regenerated and control mother plants (Fig. 7A, B), thus confirming genetic stability. The Jaccard’s similarity index based dendrogram of mother plant as well as tissue culture derived plantlets is presented in Fig. 8.

**Table 4:** List of scot primers, their sequences, %G/C, tm, number of bands and their approximate band length (bp) obtained in somatic embryo derived plantlets of *C. asiaticum* L

S.No	Primer Name	Primer Sequence (5´−3´)	%G/C	Tm (°C)	Number of Bands Amplified		Band length of amplicons (bp)
1	SCoT3	CAACA**ATG**GCTACCACCG	56	48 °C		9	300–1400
2	SCoT6	CAACA**ATG**GCTACCACGC	56	54 °C		6	200–800
3	SCoT12	ACGAC**ATG**GCGACCAACG	61	58 °C		4	300–1000
4	SCoT18	ACC**ATG**GCTACCACCGCC	67	50 °C		8	300–1200
5	SCoT23	CACC**ATG**GCTACCACCAG	61	50 °C		9	200–1200
6	SCoT33	CC**ATG**GCTACCACCGCAG	67	58 °C		8	250–1500

### GC-MS analysis

**Fig. 9:**
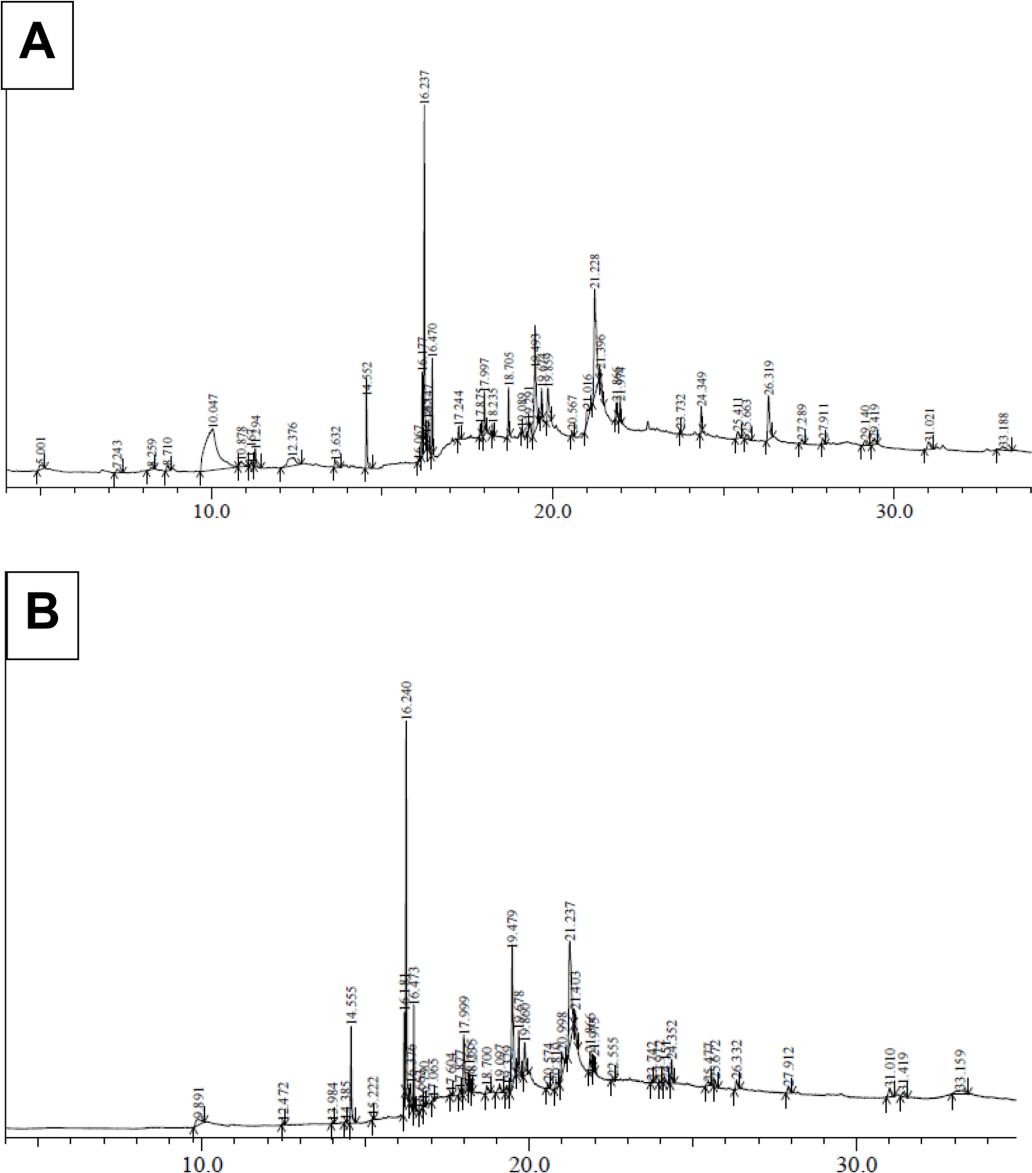
GC-MS chromatograms of methanolic leaf extracts of (**A**) in vivo grown *C. asiaticum* L. plants, and (**B**) in vitro grown *C. asiaticum* L. plants

**Fig. 10:**
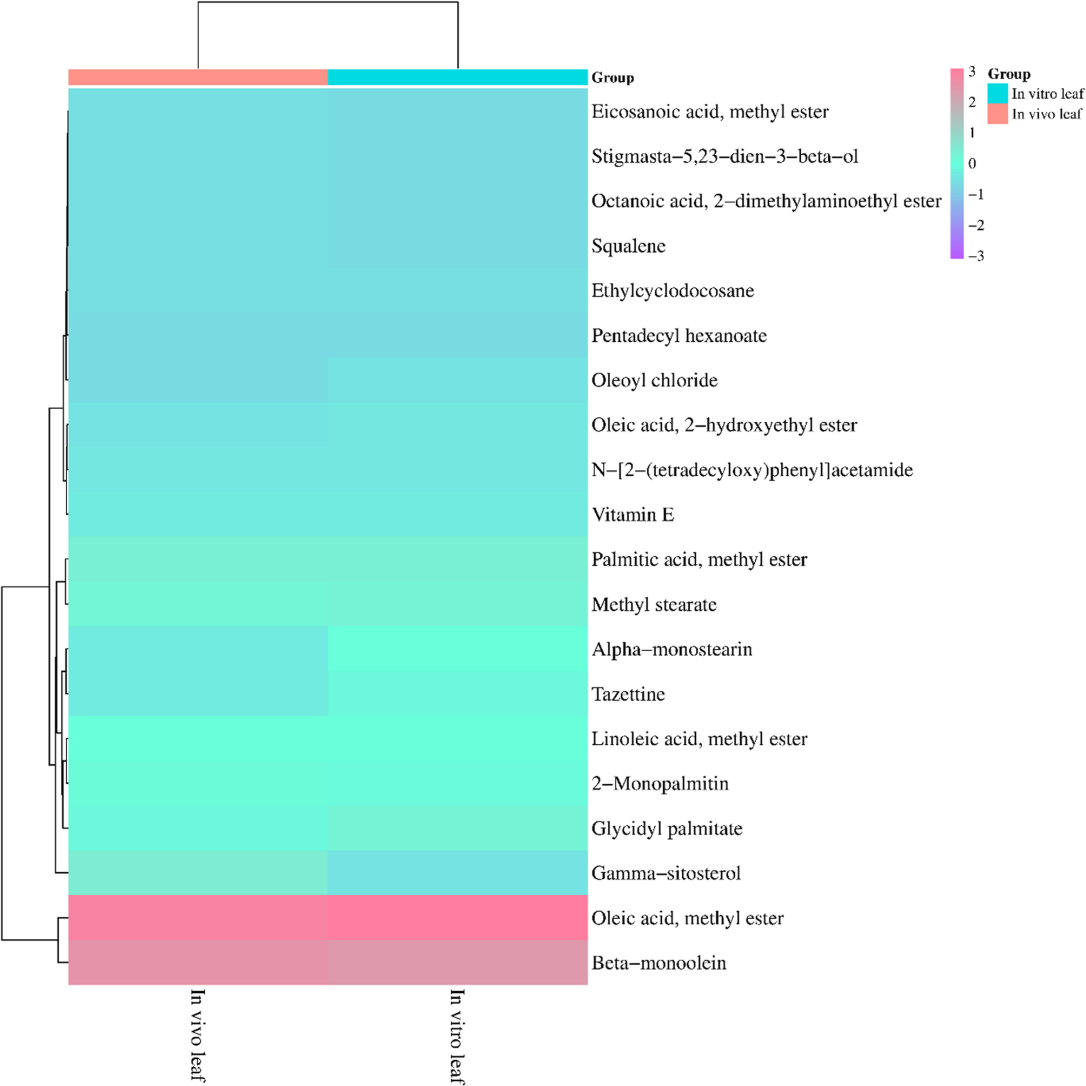
Heat map cluster analysis indicating the relative abundance of phytocompounds detected in both the methanolic leaf extracts of in vivo and in vitro raised *C. asiaticum* L. by GC-MS technique

The GC-MS analysis of methanolic leaf extracts of in vivo and in vitro (somatic embryo derived) grown *C. asiaticum* plants revealed the presence of more than 40 phytocompound peaks in the obtained total ion chromatograms (Fig. 9A, B), which were identified by assessing their retention time (RT), peak area (%), molecular formula and molecular weight as described by NIST library (Table 5). The heat map cluster analysis presented in Fig. 10 showed the relative abundance of about 20 common bioactive compounds identified in both the extracts. Some of them are tazettine, squalene, vitamin E, gamma-sitosterol, glycidyl palmitate, glycidyl oleate, alpha-monostearin, beta- monoolein, 2-monopalmitin, pentadecyl hexanoate. The phytoconstituents of field grown leaf showed compounds like guanosine (24.54%), isomenthol (1.89%), epicedrol (0.93%), phytyl stearate (0.77%), dihydro-brassicasterol (0.69%), neophytadiene (0.64%), gamma-tocopherol (0.12%). The in vitro raised leaf extract exclusively contained phytocompounds such as glycidyl oleate (13.10%), stigmasta-3,5-diene (0.35%), 1-monolinolein (0.34%), palmidrol (0.25%), dimethyl myristamine (0.16%), lauric acid methanolamine (0.08%). The presence of diverse phytocompounds in both samples, proves that *Crinum* is a good repository of pharmaceutically important compounds.

**Table 5:** Comparative GC-MS profile of methanolic leaf extracts of in vivo and in vitro (somatic embryo derived) grown *C. asiaticum* L

S.No.	Name of the compound	Retention time (min)		Peak area %		Molecular formula	Molecular weight (daltons)
		In vivo leaf	In vitro leaf	In vivo leaf	In vitro leaf		
1	2-propyltetrahydro-pyran-3-ol	5.001	-	0.74	-	C_8_H_16_O_2_	144
2	(3,3,4-Trimethyl-4-pentenyl) benzene	7.243	-	0.53	-	C_14_H_20_	188
3	3-Ethylamino-5-hexene-2-ol	8.259	-	0.23	-	C_8_H_17_NO	143
4	N-Phenethyl-2-methylbutylidenimine	8.71	-	0.53	-	C_13_H_19_N	189
5	Nitroisobutylglycerol	-	9.891	-	1.85	C_4_H_9_NO_5_	151
6	Guanosine	10.047	-	24.54	-	C_10_H_13_N_5_O_5_	283
7	N-(2-phenylethyl) pentopyranosylamine	10.878	-	1.06	-	C_13_H_19_NO_4_	253
8	1-Naphthalenemethanamine	11.167	-	0.25	-	C_11_H_11_N	157
9	Epicedrol	11.294	-	0.93	-	C_15_H_26_O	222
10	Isopropyl decanoate	12.376	-	3.08	-	C_13_H_26_O_2_	214
11	Myristic acid, methyl ester	-	12.472	-	0.18	C_15_H_30_O_2_	242
12	Neophytadiene	13.632	-	0.64	-	C_20_H_38_	278
13	1,2-Benzenedicarboxylic acid, bis(2-methylpropyl) ester	-	13.984	-	0.13	C_16_H_22_O_4_	278
14	Dimethyl myristamine	-	14.385	-	0.16	C_16_H_35_N	241
15	Palmitic acid, methyl ester	14.552	14.555	3.89	5.66	C_17_H_34_O_2_	270
16	Ethyl hexadecanoate	-	15.222	-	0.18	C_18_H_36_O_2_	284
17	11-Dodecen-2-one	16.067	-	0.08	-	C_12_H_22_O	182
18	Linoleic acid, methyl ester	16.177	16.181	2.44	3.62	C_19_H_34_O_2_	294
19	Oleic acid, methyl ester	16.237	16.24	12.62	19.16	C_19_H_36_O_2_	296
20	9-Octadecenoic acid, methyl ester	16.285	-	0.17	-	C_19_H_36_O_2_	296
21	Isomenthol	16.347	-	1.89	-	C_10_H_20_O	156
22	Linolenic acid, methyl ester	-	16.376	-	1.33	C_19_H_32_O_2_	292
23	Methyl stearate	16.47	16.473	3.33	4.98	C_19_H_38_O_2_	298
24	Lauric acid monoethanolamine	-	16.663	-	0.08	C_14_H_29_NO_2_	243
25	1-Monolinolein	-	16.79	-	0.34	C_12_H_38_O_4_	354
26	Methyl 9-cis,11-trans-octadecadienoate	-	17.065	-	0.24	C_19_H_34_O_2_	294
27	Phytol acetate	17.244	-	0.49	-	C_22_H_42_O_2_	338
28	(7E)−2-Methyl-7-hexadecene	-	17.604	-	0.19	C_17_H_34_	238
29	Octanoic acid, 2-dimethylaminoethyl ester	17.875	17.877	0.5	0.39	C_12_H_25_NO_2_	215
30	Glycidyl palmitate	17.997	17.999	1.93	5.02	C_19_H_36_O_3_	312
31	Palmidrol	-	18.165	-	0.25	C_18_H_37_NO_2_	299
32	Eicosanoic acid, methyl ester	18.235	18.235	0.43	0.51	C_21_H_42_O_2_	326
33	9-Octadecenamide	-	18.7	-	0.84	C_1_H_35_NO	281
34	2-Hexadecen-1-ol, 3,7,11,15-tetramethyl-, 1-acetate	18.705	-	1.87	-	C_22_H_42_O_2_	338
35	Oleoyl chloride	19.089	19.097	0.18	1.14	C_18_H_33_ClO	300
36	2-Formylhexadecane	19.291	-	0.34	-	C_1_H_34_O	254
37	Fumaric acid, 2-dimethylaminoethyl nonyl ester	-	19.329	-	0.76	C_17_H_31_NO_4_	313
38	(9Z)−9-Octadecenal	-	19.479	-	13.10	C_21_H_38_O_3_	338
39	Glycidyl oleate	19.493	-	7.50	-	C_21_H_38_O_3_	338
40	Alpha-monostearin	19.674	19.678	1.18	3.58	C_21_H_42_O_4_	358
41	2-Monopalmitin	19.859	19.860	2.92	3.42	C_19_H_38_O_4_	330
42	Pentadecyl hexanoate	20.567	20.574	0.26	0.38	C_21_H_42_O_2_	326
43	Methyl 4-methoxyoctadecanoate	-	20.810	-	0.66	C_20_H_40_O_3_	328
44	Tazettine	21.016	20.998	1.4	2.82	C_18_H_21_NO_5_	331
45	Beta-monoolein	21.228	21.237	11.4	15.61	C_21_H_40_O_4_	356
46	Methyl petroselinate	21.396	-	1.15	-	C_19_H_36_O_2_	296
47	Octadecanoic acid, 2,3-dihydroxypropyl ester	-	21.403	-	1.68	C_21_H_42_O_4_	358
48	Oleic acid, 2-hydroxyethyl ester	21.866	21.866	0.71	1.55	C_21_H_40_O_3_	340
49	Squalene	21.974	21.975	0.64	0.58	C_30_H_50_	410
50	n-Hexatriacontane	-	22.555	-	0.18	C_36_H_74_	506
51	gamma-tocopherol	23.732	-	0.12	-	C_28_H_48_O_2_	416
52	1-Bromotetracosane	-	23.742	-	0.15	C_24_H_49_Br	416
53	2-Methylpentacosane	-	23.972	-	0.20	C_26_H_54_	366
54	Stigmasta-3,5-diene	-	24.151	-	0.35	C_29_H_48_	396
55	Vitamin E	24.349	24.352	1.18	1.71	C_29_H_50_O_2_	430
56	Dihydrobrassicasterol	25.411	-	0.69	-	C_28_H_48_O	400
57	Methyl 18-propylhenicosanoate	-	25.477	-	0.55	C_25_H_50_O_2_	382
58	Stigmasta-5,23-dien-3-beta-ol	25.663	25.672	0.44	0.59	C_29_H_48_O	412
59	Gamma-sitosterol	26.319	26.332	4.07	1.07	C_29_H_50_O	414
60	Cycloeucalenol	27.289	-	0.36	-	C_30_H_50_	426
61	Ethylcyclodocosane	27.911	27.912	0.47	0.83	C_24_H_48_	336
62	Phytyl palmitate	29.140	-	0.49	-	C_36_H_70_O_2_	534
63	9,19-Cyclo-9-beta-lanost-24-en-3-beta-ol	29.419	-	0.56	-	C_30_H_50_O	426
64	N-[2-(tetradecyloxy) phenyl] acetamide	31.021	31.010	0.99	1.34	C_22_H_37_NO_2_	347
65	2,4,6,8-Tetramethyl-1-octacosanol	-	31.419	-	0.71	C_32_H_66_O	466
66	(22E)-Stigmasta-7,22-dien-3-yl acetate	-	33.159	-	1.95	C_31_H_50_O_2_	454
67	Phytyl stearate	33.188	-	0.77	-	C_38_H_47_O_2_	562

**Fig. 11:**
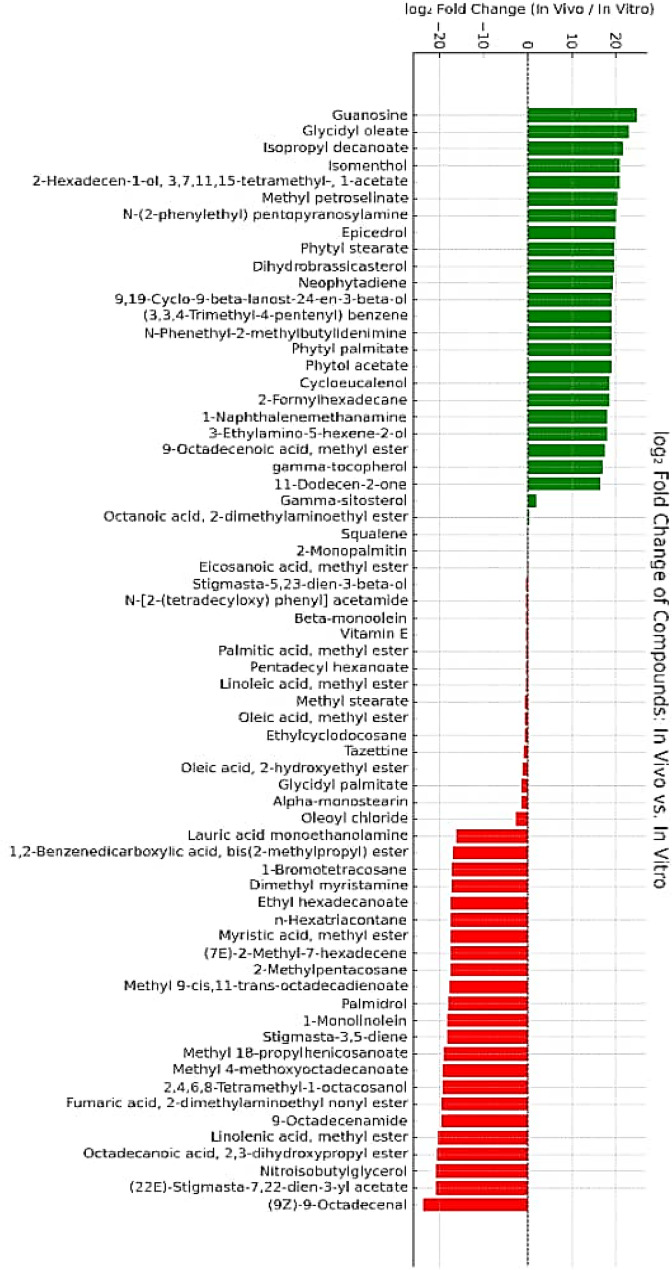
Log₂ fold change (log₂FC) in compound abundance between in vivo and in vitro leaf extracts. Each bar represents a single compound. Positive values (green bars) indicate compounds that are more abundant in in vivo sample, while negative values (red bars) indicate higher abundance in in vitro sample. A log₂FC of ± 1 corresponds to a 2-fold change in abundance

The differential abundance of metabolites is presented as log₂ fold change (log₂FC) values (Fig. 11). Several metabolites were significantly abundant in in vivo leaf sample, with the highest induction observed for guanosine, followed by glycyl oleate, isopropyl decanoate, isomenthol, and 2-hexadecen-1-ol, 3,7,11,15-tetramethyl-, 1-acetate. Additional notable metabolites included methyl petroselinate, N-(2-phenylethyl) pentopyranosylamine, epicredol, and phytol stearate. Conversely, a marked abundance was observed in compunds like (9Z)−9-octadecenal, (22E)-stigmasa-7,22-dien-3-yl acetate, nitrosobutylglycerol, octadecanoic acid, 2,3-dihydroxypropyl ester, and linolenic acid methyl ester in in vitro samples. Other metabolites included fumaric acid, 2-dimethylaminomethyl nonyl ester, 2,4,6,8-tetramethyl-1-octacosanol, and ethyl 4-methyloctadecanoate. Overall, the metabolite profile revealed a clear shift, with several lipids, fatty acid esters, and sterol derivatives being either highly induced or suppressed, indicating substantial metabolic reprogramming under the studied conditions.

## Discussion

The present investigation was conducted to optimize a reproducible and efficient in vitro plant propagation system via direct somatic embryogenesis for continuous production of medically valuable bioactive. In the current study, the effect of different concentrations and combinations of auxins and cytokinins was assessed for somatic embryogenesis and plant regeneration from bulb-scales in *C. asiaticum*. It was observed that a high cytokinin with low auxin level successfully induced more somatic embryos directly on bulb-scale surfaces than on medium added with auxin alone. The maximum somatic embryo induction frequency (95.83%) was noted on NAA (2.7 µM) and BAP (4.4 µM) fortified MS medium, post 4 weeks of inoculation. Previous reports documented similar observations where a combined influence of BAP and NAA promoted somatic embryogenic potential in several monocotyledons (*Fritillaria meleagris*, *Oryza sativa* L.) [28, 29] and dicot plants like *Cicer arietinum* cv. Bivanich and *Santalum album* L [30, 31]. A combination of 2,4-D and BAP was also noted to be promotive for indirect somatic embryogenesis in *Crinum malabaricum* [32]. Auxins are believed to be involved in induction and multiplication of embryos (both direct and indirect ways), but obstruct subsequent development and maturation [33]. Cytokinins, on the other hand, are associated with cell divisions [34], thus may release tension caused by auxin in later developmental stage of embryos. Histological and SEM analysis of tissues confirmed the origin and different developmental stages of somatic embryos in culture. Histological investigations revealed direct origin of somatic embryo from the bulb-scale surface i.e. epidermal cellular region and, thus this technique has widely been used in studying embryo characteristics in various studied plants like *Oryza sativa* L [35]. and *Coffea arabica* L [36]. The maturation and conversion of somatic embryos into shoots were achieved on BAP fortified MS medium within 3 weeks of inoculation. Similar stimulatory effects of BAP on somatic embryo maturation and conversion were noted in several plant species [37, 38]. The efficient root induction is a critical step in establishing a successful in vitro plant propagation protocol. For rooting, three different auxins, namely NAA, IBA and IAA were used at various concentrations. Among all the auxins tested, NAA (5.4 µM) showed better root induction and development frequency of 91.67%, followed by IBA and IAA treatments. Govindaraju and Arulselvi [39] attributed NAA’s importance over IAA and IBA in rooting to the fact that NAA is not degraded by auxin oxidases in medium and thus may be a suitable choice for in vitro rooting programme. Several earlier reports proved positive influence of NAA on rooting in different plants like *Syzygium cumini* L [40]., *Broussonetia papyrifera* [41], *Ungernia victoris* Vved. EX Artjush [42]. The well rooted plants were later transferred to plastic pots filled with soil (1): soilrite (1) before transfer to outdoor condition. The plants grew normally as there was higher level of CO_2_ and more light which encourage photosynthesis and ensure better plantlets’ survival ability under in vivo condition [43].

The occurrence of somaclonal variations (genetic instability) in tissue culture raised plants are not new and this occurs due to unforced stress caused by factors like continuous involvement and pressure of various PGRs, multiple subculturing and long exposure to in vitro physical environment [42, 44]. Therefore, it is essential to ascertain the genetic stability of micropropagated plants and it can be assessed through cytological, biochemical and molecular marker systems. The cytological status of in vitro regenerated plants’ root tips was performed and compared with mother or donor plants; all the regenerated and mother plants showed an equal number of chromosomes (2n = 44), revealing cytological stable status with no change in ploidy numbers. The chromosomal analysis has frequently been conducted in investigating chromosomal stability in in vitro regenerated populations of various plant species [12, 45]. Flow cytometry method (FCM) is also an intriguing approach to determine the genetic homogeneity of regenerated plants by comparing with mother plants in measuring relative genome size [46]. The flow cytometry results of our study demonstrated that the fluorescence peaks of PI-stained nuclei of regenerated plants were identical to the peaks of donor (mother) plants, implying no difference in ploidy of regenerated plants (31.79 pg DNA) with respect to mother plant (31.51 pg DNA). Multiple studies reported the analysis of ploidy by using flow cytometry in several members of Amaryllidaceae (monocot) such as *Lycoris spp*., *Zephyranthes spp.*,* Allium cepa* [11, 47] as well as in other dicotyledonous plants like *Cucumis melo* [48], *Ficus carica* [49] and *Brassica juncea* [50]. To validate the genetic uniformity of the laboratory raised plants further, SCoT marker-based PCR method was undertaken. Among different PCR based markers, SCoT molecular marker has recently been gained immense popularity in investigating genetic homogeneity and diversity in various plant species [51] as it targets a short-conserved region flanking ATG start codon, present in plant genome [17]. The current study employed six SCoT primers, revealing 92.2% monomorphism between somatic embryos derived plants and mother (control) plants. This suggests that the in vitro regenerated *C. asiaticum* plants were identical to corresponding mother plant with little or no genetic variation. Earlier, the genetic uniformity of somatic embryo derived plants of *Crinum malabaricum* [32] and *Crinum brachynema* [52] were also checked by SCoT marker analysis showing monomorphism and is being regarded as a more authenticated method in evaluating genetic fidelity of micropropagated plants than the other available markers. There are several factors like explant type, genotype, chemical composition of media, light conditions etc., are believed to influence genetic variability during culture [53, 54].

The primary objective of in vitro culture technology is to propagate plants and to extract high quality medically important phyto-compounds for pharmaceutical purposes [16]. Keeping the importance in mind, we studied metabolite profiles of mother (control) and micropropagated plants of *C. asiaticum* by using GC-MS. The methanolic extracts of both samples had a wide range of compounds such as alkaloids, fatty acids, sterols, triterpenoids etc. A total of 20 phytocompounds like tazettine, squalene, vitamin E, gamma-sitosterol, linoleic acid, eicosanoic acid, oleic acid, 2-monopalmitin, alpha-monostearin etc. were identified in both the samples (Table 5). Tazettine, an important alkaloid of Amaryllidaceae, is synthesized from precursor molecule pre-tazettine and has found applications in antitumor and antiviral activity [55]. Squalene is a triterpenoid, earlier shown anti-oxidative, anti-cancerous, anti-atherosclerotic and hepatoprotective activities [56]. Beta-sitosterol, previously noted to be active against several biological activities such as anti-microbial, anti-inflammatory, anti-cancerous, anti-diabetic properties and was reported to be present in a number of plants [57]. Likewise, fatty acids have been playing a critical role in an organism’s life by preventing and treating diseases. Linoleic acid and oleic acid are said to be involved in cholesterol level reduction, lowering coronary heart disorder incidences, heart stroke and Alzheimer disease [58]. Eicosanoic acid, another fatty acid member, is noted active in various biological functions as anti-mutagenic and anti-oxidant agents [59]. There are several phytoconstituents, exclusively present to each sample at variable quantities and this may be attributed to the fact that the synthesis of bioactive is also influenced by several external factors like plant growth regulators uses, medium composition and other cultural/physiological conditions [21]. Similar secondary metabolite profiling by GC-MS technique has been carried out in other Amaryllidaceae members such as *Caliphruria tenera* Baker [60] and *Narcissus tazetta* var. chinensis [61]. Thus the regenerated *C. asiaticum* plant is proved to be a potent, consistent source of therapeutic compounds. However, one inherent limitation of GC–MS is its applicability solely applied to the separation and identification of low molecular weight (approximately 50–600 Da) and volatile compounds, and thus needs further integrated studies to isolate and identify each individual bioactives from plant extracts.

## Conclusion

The present study serves as a report on induction of direct somatic embryogenesis in *C. asiaticum* using bulb-scale explants. Histological and SEM analyses confirmed somatic embryo formation directly on bulb-scale surfaces. The chromosome number, 2 C DNA content and SCoT molecular marker confirmed that the embryo derived plants were true-to-type. GC-MS analysis revealed that in vitro obtained plants possess many metabolites (if not all), similar to that of mother (donor) plant which proves to be a suitable alternative source for secondary metabolites. Based on our findings, this optimized protocol can be beneficial to various industries for the large-scale production of important metabolites without any alteration in their genomic make-up.

## Materials and methods

### Collection of plant material and surface sterilization of explants

Healthy bulbs of *Crinum asiaticum* L. were procured from the Herbal Garden, Jamia Hamdard, New Delhi, India (28° 30’ 48.768” N latitude and 77° 14’ 54.81” E longitude). The bulb explants were initially soaked in detergent Teepol for 20 min and were then thoroughly washed under running tap water for 30 min. Thereafter, the bulbs were rinsed with 70% ethanol for 6 min and further treated with 0.1% mercuric chloride (SRL, Mumbai, India) solution for 2–3 min. Finally, the bulbs were washed thrice with sterilized distilled water to eliminate the residual disinfecting agents.

### Culture medium and incubation conditions

The surface sterilized bulbs were used as explants and were cut into small segments (4–5 cm). The explants were inoculated on Murashige and Skoog (MS) basal medium [62] supplied with 3% (w/v) sucrose (Himedia, Mumbai, India) and 0.8% (w/v) agar (Himedia, Mumbai, India). The pH adjustment of the medium was made at 5.7 and autoclaved at 121 °C for 15–20 min. The culture room was maintained at 24 ± 2 °C temperature, 16 h illumination photoperiod (irradiance of 50 µmol m^−2^ s^−1^) and a relative humidity of 55–60% for 2 months.

### Direct somatic embryogenesis

The disinfected individual bulb-scales were inoculated in MS medium fortified with different concentrations of 2, 4-D, NAA and BAP (alone or combined) for induction and development of somatic embryos. The somatic embryogenic frequency as well as the mean number of somatic embryos per explant were recorded post 4 weeks of culture period. The somatic embryos were later transferred for maturation and conversion to plantlets on varied concentrations of BAP and the frequency was noted after three and six weeks of culture.

### Histological and scanning electron microscopic (SEM) study

For histological study, the explants bearing somatic embryos were primarily fixed with FAA (formalin: acetic acid: ethanol) solution in 1:1:3 ratio (*v: v: v*) for 24 h, dehydrated in an increasing ethanol graded series and finally embedded in saturated paraffin wax [63]. Fine sections (about 8–9 μm) of embedded tissues were cut on a rotary microtome (Spencer, USA) and mounted on a clean glass slide. The tissues were then dewaxed with xylene, followed by using 5% hematoxylin and 2% eosin dyes. After staining, the sections were visualized with the help of a light microscope (Nikon Optiphot, Tokyo, Japan).

To study the developmental stages of somatic embryos at ultra-structural levels, SEM analysis was conducted. The explant with somatic embryos were initially fixed using a primary fixative (Karnovsky’s fixative), followed by washing with 0.1 M phosphate buffer at 4 °C. After that, the samples were dehydrated with an increasing acetone graded series (30–100%) at 15 min intervals and dried at the critical point (1100 psi) for 25–30 min. The dried samples were sputter-coated with gold and visualized under a scanning electron microscope (Zeiss, Oberkochen, Germany) at a voltage of 20 kV.

### Root induction and acclimatization

The somatic embryo derived shoots were cultured onto full MS medium augmented with three auxins, namely indole-3-acetic acid (IAA)/indole-3-butyric acid (IBA)/NAA (Sigma-Aldrich, Missouri, USA) at variable concentrations. The root induction frequency (%) and the mean root numbers per shoot were noted after 4 weeks of culture. After removing the agar, the in vitro derived plants (total count = 24) of *C. asiaticum* with well-developed and healthy roots were transferred into pots containing soil and soilrite in the ratio of 1:1 (w: w), wrapped with transparent polybags to maintain humidity. Later on, these plantlets were allowed to grow under greenhouse environment at 26 ± 2 °C temperature for 10–12 h photoperiod with a relative humidity of 70–80%.

### Cytological analysis

The cytological study of the samples was conducted following the protocol of Malik et al. [64]. Young root tips (3–4 each) of randomly selected in vitro (somatic embryo derived) and in vivo grown plants of *C. asiaticum* were collected, washed thoroughly with double distilled water and placed in 0.05% colchicine solution (Sigma-Aldrich, Missouri, USA) for 5 h at 4 °C. The root tips were then rinsed off with distilled water to remove colchicine remnants and fixed in aceto-alcohol solution for 2–3 h. The fixed root apices were transferred to glass vials containing aceto-orcein (1%) and HCl in a ratio of 9:1 and kept for 45 min. Finally, the stained root tips were placed on a clean slide with a cover slip, squashed and the chromosomes were counted and viewed under a light microscope (40X).

### Flow cytometry

To determine the genetic fidelity of *C. asiaticum* regenerants, healthy young leaves of somatic embryo derived plants as well as the mother (control) plants were taken for flow cytometric analyses. As a reference standard (Fig. 6A), *Zephyranthes grandiflora* with known 2 C DNA content was used in this experiment [65]. According to protocol reported by [66], about 120 mg of young leaves of each sample were chopped with sharp blades in a 1500 µL pre-chilled Galbraith’s buffer [20 mM MOPS (3-(N-morpholino)propanesulfonic acid), 30 mM sodium citrate, 45 mM MgCl_2_ and 0.1% Triton-X] for nuclei isolation. The homogenates were then filtered using a 30 μm nylon mesh and subsequently stained with 50 µg/ml PI RNase (propidium iodide RNase) (Sigma Aldrich, USA) for 8–10 min. Later on, the samples were incubated in darkness for about 1 h at 5 °C. Lastly, all the samples were analysed using a flow cytometer (BD FACS Lyric, BD Biosciences, USA) with three replicates of each sample to determine the mean DNA fluorescence intensity. The relative 2 C DNA content of *C. asiaticum* plants was calculated according to the below formula [67]:

$$\begin{aligned}\:2\mathrm{C}\:\mathrm{D}\mathrm{N}\mathrm{A}\:\mathrm{c}\mathrm{o}\mathrm{n}\mathrm{t}\mathrm{e}\mathrm{n}\mathrm{t}\:\mathrm{o}\mathrm{f}\:\mathrm{s}\mathrm{a}\mathrm{m}\mathrm{p}\mathrm{l}\mathrm{e}\:\left(\mathrm{p}\mathrm{g}\right)&=2\mathrm{C}\:\mathrm{D}\mathrm{N}\mathrm{A}\:\mathrm{c}\mathrm{o}\mathrm{n}\mathrm{t}\mathrm{e}\mathrm{n}\mathrm{t}\:\mathrm{o}\mathrm{f}\:\mathrm{s}\mathrm{t}\mathrm{a}\mathrm{n}\mathrm{d}\mathrm{a}\mathrm{r}\mathrm{d}\:\left(pg\right)\times\\&\:(\mathrm{m}\mathrm{e}\mathrm{a}\mathrm{n}\:\mathrm{p}\mathrm{o}\mathrm{s}\mathrm{i}\mathrm{t}\mathrm{i}\mathrm{o}\mathrm{n}\:\mathrm{o}\mathrm{f}\:\mathrm{G}0/\mathrm{G}1\:\mathrm{p}\mathrm{e}\mathrm{a}\mathrm{k}\:\mathrm{o}\mathrm{f}\:\mathrm{s}\mathrm{a}\mathrm{m}\mathrm{p}\mathrm{l}\mathrm{e})/\\&(\mathrm{m}\mathrm{e}\mathrm{a}\mathrm{n}\:\mathrm{p}\mathrm{o}\mathrm{s}\mathrm{i}\mathrm{t}\mathrm{i}\mathrm{o}\mathrm{n}\:\mathrm{o}\mathrm{f}\:\mathrm{G}0/\mathrm{G}1\:\mathrm{p}\mathrm{e}\mathrm{a}\mathrm{k}\:\mathrm{o}\mathrm{f}\:\mathrm{s}\mathrm{t}\mathrm{a}\mathrm{n}\mathrm{d}\mathrm{a}\mathrm{r}\mathrm{d})\end{aligned}$$

### DNA extraction and genetic fidelity analysis by scot molecular marker

To examine the genetic fidelity through SCoT marker, five somatic embryo derived plants of *C. asiaticum* were randomly chosen along with the mother plant. The total genomic DNA was isolated from 100 mg leaves of each sample by the cetyltrimethyl ammonium bromide (CTAB) method [68]. The isolated DNA was assessed for its quantity and quality by using 0.8% agarose gel electrophoresis. A total of 10 SCoT primers were initially used, out of which six primers generated reproducible and scorable bands and were used to conduct PCR amplification reactions. The SCoT-PCR amplification was executed with a total volume of 20µL containing 50 ng of total genomic DNA, 2.5 mM MgCl_2_, 10 mM dNTPs, 10X Taq polymerase buffer, SCoT primers (18 nucleotides long), 5 units/µL of Taq DNA Polymerase (Sigma-Aldrich) and sterile distilled water. The amplifications were done in a thermal cycler (Bio-Rad, USA) with an initial denaturation of DNA at 94 °C for 5 min, followed by 35 cycles of 30 s denaturation at 94 °C, 30 s annealing at 50 °C, followed by 1 min extension at 72 °C with a final extension set at 72 °C for 5 min. The amplified PCR products were subject to electrophoresis on 1.5% agarose gel using 1X TBE (Tris HCl, Boric acid, EDTA) buffer and all amplification reactions were repeated thrice for ensuring the reproducibility of the bands. The gel photographs were taken using a gel documentation system (Bio-Rad, USA). The sizes of the amplicons were determined by 1 kb DNA ladder (Thermo Scientific, USA). The Jaccard’s similarity coefficient was used to calculate the genetic similarity (GS) values between the mother plant and the plantlets, grown in vitro. After obtaining the similarity coefficients, a dendrogram was created using the UPGMA (Unweighted Pair Group Method of Arithmetic Averages) [69] method in NTSYSpc software (version 2.02, Rohlf, New York, USA) [70].

### Metabolomics study via GC-MS technique

#### Preparation of leaf extracts

The samples were prepared according to the protocol described by Hussain et al. [21]. Healthy leaf samples from in vivo and in vitro (before acclimatization stage) raised *C. asiaticum* plants were collected, shade dried and pulverized into fine powder using mortar and pestle. Each powdered sample was extracted individually with absolute methanol (HPLC grade) as a solvent using Soxhlet apparatus (Glasscolabs, India). These samples were evaporated to dryness at room temperature for 72 h with the help of aluminium foil. All the methanolic extracts were properly labelled and stored in airtight glass vials at 4 °C till further use for GC-MS analysis.

#### GC-MS analysis

The gas chromatography coupled with mass spectrometry (GC-MS) analyses of methanolic leaf extracts of in vivo and in vitro plants of *C. asiaticum* were performed on GC–MS-QP-2010 plus equipment (Shimadzu, Japan) having following program settings: Pure helium gas (99.99%) was used as a carrier gas at a constant flow rate of 1.21 ml/min with an inlet pressure of 90.5 kPa and injection volume of 2µL (split ratio 10:1) was applied. The ion source and injector temperature were maintained at 220 °C and 260 °C, respectively. The column oven temperature was programmed from 100 °C to 300 °C (hold time of 17 min). The mass spectra were obtained at 70 eV with a scan time of 0.2 s for fragments ranging from 40 to 600 m/z, the GC-MS running time for both the samples were 35–40 min. The relative area percentage of each bioactive compound was measured by comparing its mean peak area with all the total areas. Identification of phytocompounds was done on the basis of retention time, peak area, molecular formula as well as comparison of mass spectra of unknown compounds with mass spectral patterns of phytocompounds already stored in the NIST17 (National Institute of Standards and Technology) library database.

### Statistical analysis

All the in vitro based experiments were conducted with five replicates (one explant per test tube) and each experiment was repeated thrice. Each measured parameter was subject to one way analysis of variance (ANOVA) and the mean comparisons were estimated by Duncan’s multiple range test (DMRT) at *p* < 0.05 level [71] using SPSS software (version 16, SPSS Inc., Chicago, USA). The obtained data were expressed as mean value ± standard error (SE). The heatmap analysis was performed by using R programming software (ver 4.3.3) to study the relative abundance of metabolites (unit variance scaling) in samples.

## Data Availability

Yes, I have research data to declare.

## Abbreviations

ANOVA: Analysis of variance
BAP: 6-Benzylaminopurine
DMRT: Duncan’s Multiple Range Test
FCM: Flow Cytometry Method
GC-MS: Gas Chromatography-Mass Spectrometry
IAA: Indole-3-acetic acid
IBA: Indole-3-butyric acid
MS: Murashige and Skoog
NAA: α-naphthalene acetic acid
